# MULTILEVEL SOCIAL FACTORS OF HEALTH AND AGING AMONG DIVERSE POPULATIONS

**DOI:** 10.1093/geroni/igad104.2007

**Published:** 2023-12-21

**Authors:** Agus Surachman, Jourdyn Lawrence, Rose Ann DiMaria-Ghalili

## Abstract

While a large body of evidence increasingly documents how individual-level modifiable risk factors shape where and how people age, the aspects of the social environment in which people grow older deserve more focus. Here, we aim to identify examples of multilevel social factors that shape health and aging among samples from diverse populations and datasets. The overarching goal of this work is to yield evidence of the impact of social factors on healthy aging, specifically focusing on cognitive functioning, physical activity, unmet needs, and psychological distress for middle-aged and older adults. Our panel includes four studies focusing on distinct aspects of the social environment and outcomes that affect healthy aging. Specifically, the first presentation uses instrumental variable analysis to estimate a causal association between racial discrimination within institutional settings (e.g., housing, employment) and cognitive function measures. The second study considers neighborhood deprivation (measured using the area deprivation index and personal belief in the neighborhood) as a structural context that may strongly limit adults’ engagement in daily physical health. The third presentation identifies an association between the home environment (i.e., home disorder and disrepair) and unmet care needs (e.g., going without needed assistance with everyday activities such as eating or bathing). Our last presentation shows relationships between measures of economic status (e.g., food insecurity), social support impact, and psychological distress. We close by integrating critical findings from each study and consider the implications of each study on future research and interventions.

